# Supplementary material for: A Critical View of the Application of the APEX Software (Aqueous Photochemistry of Environmentally-Occurring Xenobiotics) to Predict Photoreaction Kinetics in Surface Freshwaters
Source: Molecules. 2019 Dec 18;25(1):9. doi: 10.3390/molecules25010009 (PMC7017383; doi:10.3390/molecules25010009)
Supplement: Supplementary file 1 [file molecules-25-00009-s001.zip › 3.2.4_gcc-4.4.0.zip!/3.2.4_gcc-4.4.0/3.2.4_gcc-4.4.0/doc/octave/html/interpreter/Models.html]

Models - Untitled


Next: Distributions,
Previous: Tests,
Up: Statistics


---

### 25.5 Models

— Function File: [theta, beta, dev, dl, d2l, p] = **logistic\_regression** (y, x, print, theta, beta)  
> Perform ordinal logistic regression.
>
> Suppose y takes values in k ordered categories, and let
> `gamma_i (`x`)` be the cumulative probability that y
> falls in one of the first i categories given the covariate
> x. Then
>
> ```
>           [theta, beta] = logistic_regression (y, x)
> ```
>
> fits the model
>
> ```
>           logit (gamma_i (x)) = theta_i - beta' * x,   i = 1 ... k-1
> ```
>
> The number of ordinal categories, k, is taken to be the number
> of distinct values of `round (`y`)`. If k equals 2,
> y is binary and the model is ordinary logistic regression. The
> matrix x is assumed to have full column rank.
>
> Given y only, `theta = logistic_regression (y)`
> fits the model with baseline logit odds only.
>
> The full form is
>
> ```
>           [theta, beta, dev, dl, d2l, gamma]
>              = logistic_regression (y, x, print, theta, beta)
> ```
>
> in which all output arguments and all input arguments except y
> are optional.
>
> Setting print to 1 requests summary information about the fitted
> model to be displayed. Setting print to 2 requests information
> about convergence at each iteration. Other values request no
> information to be displayed. The input arguments theta and
> beta give initial estimates for theta and beta.
>
> The returned value dev holds minus twice the log-likelihood.
>
> The returned values dl and d2l are the vector of first
> and the matrix of second derivatives of the log-likelihood with
> respect to theta and beta.
>
> p holds estimates for the conditional distribution of y
> given x.
